# Aggressive Antisocial Behaviors Are Related to Character Maturity in Young Swedish Violent Offenders Independent of ADHD

**DOI:** 10.3389/fpsyt.2016.00185

**Published:** 2016-11-16

**Authors:** Thomas Nilsson, Örjan Falk, Eva Billstedt, Nóra Kerekes, Henrik Anckarsäter, Märta Wallinius, Björn Hofvander

**Affiliations:** ^1^Department of Forensic Psychiatry, Institute of Neuroscience and Physiology, University of Gothenburg, Gothenburg, Sweden; ^2^Centre for Ethics, Law and Mental Health, University of Gothenburg, Gothenburg, Sweden; ^3^Gillberg Neuropsychiatry Centre, Institute of Neuroscience and Physiology, University of Gothenburg, Gothenburg, Sweden; ^4^Department of Health Sciences, University West, Trollhättan, Sweden; ^5^Child and Adolescent Psychiatry, Faculty of Medicine, Department of Clinical Sciences, Lund, Lund University, Lund, Sweden; ^6^Research and Development Unit, Regional Forensic Psychiatric Clinic, Växjö, Sweden

**Keywords:** young male criminal offenders, character maturity, ADHD, criminality, aggression, psychopathic traits

## Abstract

**Background:**

Antisocial personality and psychopathic traits have constantly been found to accompany criminal and aggressive behaviors, but little attention has been given to aspects of character maturity and its relation to such behaviors. The present study investigated (1) whether level of character maturity (low, medium, and high) is associated with amount of aggressive antisocial behaviors (AABs) and psychopathic traits in young men imprisoned for violent criminality, and (2) whether such an association is independent of coexisting attention-deficit hyperactivity disorder (ADHD).

**Methods:**

Swedish males (*N* = 270, aged 18–25) sentenced to prison for violent and/or sexual criminality in the western region of the Swedish Prison and Probation Service underwent a thorough clinical examination during their incarceration. Data on character maturity, as measured by the character dimensions Self-Directedness and Cooperativeness of the Temperament and Character Inventory, were available for *n* = 148 subjects and were used to divide these offenders into three groups with low, medium, and high character maturity. These groups were then compared for variables reflecting criminal history, a DSM-IV diagnosis of ADHD, conduct disorder (CD) and substance use disorders (SUD), aggressive behaviors, and psychopathic traits.

**Results:**

Character maturity was consistently associated with less AABs and psychopathic personality traits; the group with the highest character maturity showed: (i) a later age at onset of criminality, (ii) a smaller number of prior violent criminal acts, (iii) lower prevalence of ADHD, CD, and SUD, (iv) less self-rated and expert-rated aggressive behaviors, and (v) less psychopathic traits. The association between character maturity and aggressive behaviors/psychopathic personality traits remained even when ADHD was controlled for. The only exception was sexual criminality, where the group with the highest character maturity contained the largest amount of sexual offenders.

**Conclusion:**

Higher character maturity appeared to be a protective factor among young male violent offenders, associated with less AABs, suggesting that character maturity is a promising target for treatment interventions for this group of individuals.

## Introduction

Criminologists have, during most of the twentieth century, been skeptical about the role of personality, that is, of characteristic patterns of thinking, feeling, and behaving in aggressive antisocial behavior (AAB). However, this started to change during the end of this century when scientists like Moffitt (1) presented her taxonomy of AAB where temperament plays an important role in the etiology of life-course-persistent AAB, and Gottfredson and Hirschi (2) admitted that their concept of low self-control is in accordance with what is meant by a personality trait.

### Cloningers Temperament and Character Model and Aggressive Antisocial Behaviors

A further establishment of the role of personality for the development of AAB was seen when Miller and Lynam (3) presented their meta-analytic review, where they showed that structural models of personality consistently stood at least in a moderate relation to AAB. One of the structural theories that, according to their review, was related to AAB was Cloninger’s seven factor temperament and character model measured by the Temperament and Character Inventory (TCI) (4). This psychobiological model consists of four temperament dimensions: Novelty Seeking, Harm Avoidance, Reward Dependence, and Persistence, and three character dimensions: Self-Directedness, Cooperativeness, and Self-Transcendence. All four temperament dimensions are assumed to be quite stable and genetically founded, reflecting individual differences in procedural memory and learning, which, according to Cloninger (5), can be described as an individual’s automatic associative responses to emotional stimuli determining the orientation of that individual’s habits and moods. The three character dimensions are, on the other hand, reflecting the effects of sociocultural learning and the maturational processes, and thus, an individual’s propensity to become self-aware with voluntary intensions and attitudes, or in other words, an individual’s path toward a mature character. Self-Directedness comprises the concept of the self as an autonomous individual, and it includes notions about feelings of personal integrity, self-esteem, honor, effectiveness, and a sense of direction of one’s life. Cooperativeness on the other hand is based on the concept of the self as an integral part of the human society, and it comprises feelings of affinity with the community, compassion, conscience, and a will to share with others. Together, these two dimensions constitute what could be described as the core of character maturity, that is, an essential and balanced conceptualization of self and other. According to Cloninger and coworkers (6), the configuration of temperament and its relation to character development have a strong association to personality disorders listed in the DSM-IV (7), where low scores on the character dimensions are a general sign of a personality disorder (i.e., a low level of character maturity) while the temperament configuration defines the type of disorder (i.e., conflicting and burdening temperament configurations). Those who have high Novelty seeking and low Cooperativeness (poor impulse control and lack of empathy) are more likely to also meet the criterion for a cluster B personality disorder (antisocial, narcissistic, and borderline).

In the Miller and Lynam (3) review, the strongest relation between AAB and the temperament dimensions were found for Novelty Seeking (*r* = 0.34) followed by Reward Dependence (*r* = −0.12), while the other two temperaments showed a very weak association with AAB. Among the three character dimensions, Self-Directedness and Cooperativeness showed the strongest relation to AAB (*r* = −0.25 for both), while Self-Transcendence emerged as weakly associated with AAB. Thus, the Miller and Lynam (3) review gave some empirical support for an association between a temperament constellation of impulsive and exploratory traits in combination with a weak and immature character (i.e., a character distinguished by fragile, unreliable, purposeless as well as critical, unhelpful and revengeful traits) and the development of AAB.

Some recent studies have added further support to the relation between AAB and temperament and character according to TCI, especially with regard to offenders with psychopathy. Snowden and Gray (8) found, in a sample of 121 serious and repeat offenders, that high total psychopathy checklist-revised (PCL-R) scores were associated with high Novelty Seeking and low Harm Avoidance and Cooperativeness. A related pattern was found in a Turkish study comparing 68 males with an antisocial personality disorder (ASPD) and 65 healthy controls (9). The control group showed higher scores than the ASPD group for Reward Dependence, Persistence, Self-Directedness, and Cooperativeness, while the opposite was found for Novelty Seeking and Harm Avoidance. When analyzing correlations for the whole group, PCL-R Factor 1, Factor 2, and total scores were positively associated with temperament dimensions Novelty Seeking and, somewhat surprisingly, Harm Avoidance and negatively with Reward Dependence and Persistence, as well as with the character dimensions of Self-Directedness and Cooperativeness. Common for both these studies is a pattern of high Novelty Seeking and low Cooperativeness in subjects with psychopathy, while the contradictory results for Harm Avoidance (i.e., negatively related to psychopathy in the Snowden and Gray (8) study but positively in the Basoglu and coauthors (9) study) most probably depends on sample differences.

A rather recent study on 122 male juvenile offenders (10) presented findings similar to the Snowden and Gray (8) study, showing that Psychopathy Checklist: Youth Version total scores were positively associated with Novelty Seeking and negatively with Harm Avoidance and Cooperativeness. Since this pattern is found in both juvenile and adult offenders, even if it is based on a relatively limited number of data sets, it seems reasonable to hypothesize that these temperament and character aspects are related to the development of AAB and criminality. However, Self-Directedness, which according to Cloninger (5) is somewhat of a main character dimension where low scores signals immaturity and the occurrence of a personality disorder in adults, did not emerge as associated with neither AAB nor psychopathy except for in the Basoglu and coauthors (9) study, where low scores were strongly associated with PCL-R total scores. We are, thus, in need of further studies investigating whether character maturity such as Self-Directedness is related to psychopathy and AAB.

### Childhood Onset Attention-Deficit Hyperactivity Disorder, Aggressive Antisocial Behaviors, and Character Maturity

The trajectory for individuals entering a path toward a persistent criminal life style is fairly well investigated, and some of the most important risk factors for the development are occurrence of a diagnosis of attention-deficit hyperactivity disorder (ADHD) during childhood, conduct disorder (CD), and/or use of alcohol and other illegal substances in adolescence, often combined with an early debut in criminal activity (11, 12). According to the Hofvander and coauthors’ (12) review on AAB from childhood to adulthood, about half of all children with a combined problem constellation of ADHD followed by oppositional defiant disorder (ODD) and CD with a debut before puberty develops an ASPD in adulthood. Children with ADHD have also been found to be high in Novelty Seeking and low in Self-Directedness and Cooperativeness (13), while adult psychiatric patients with high scores in life history of aggression (LHA) have been distinguished by childhood ADHD, CD, and low Cooperativeness (14).

The role of the TCI character dimensions in the problem constellation of childhood onset ADHD and behavior problems within the domain of ODD/CD is, however, not fully elucidated. Two studies have reported contradictory results. Drechsler et al. (15) found support for low Cooperativeness being related to ODD/CD (15), whereas Melegari et al. (16) reported that similarities and differences between children with ADHD or ODD was solely found within temperament dimensions (16). On the other hand, a study of 188 Russian male juvenile delinquents found that Self-Directedness and Cooperativeness were negatively correlated with amount of psychological symptoms and levels of AABs (17).

Widespread deficits in character maturity have also been seen in a Swedish study of adult patients with current and lifetime ADHD and/or autism spectrum disorders (ASDs), since extremely low scores were found for both Self-Directedness and Cooperativeness (18). Divergent findings on character dimensions in groups with neuropsychiatric problems and/or AAB seem to be a rule rather than an exception and, thus, probably reflects the complex variation in character maturity among the studied individuals. A plausible interpretation of these differences would be that there is no direct relation between the TCI character dimensions of Self-Directedness and Cooperativeness and, for example, AAB. Instead of viewing character maturity as a trait that is static in its relation to behaviors or diagnoses, it seems more in line with actual data to view it as a dynamic trait that varies among individuals in its relation to behavior domains, such as AAB, to diagnostic entities (e.g., ADHD or ODD), and over time (e.g., both at different ages within individuals and between individuals at the same ages).

Self-Directedness and Cooperativeness have also been used in combination as an overall measure for character maturity, usually in the form of total number of SDs over or below the normal zone. In the Kerekes and coauthors’ (13) study, for example, such an overall measure was used and defined in the following way: scores within 1 SD below or above the mean were considered to designate a normal character maturity, while scores below this range indicated an immature character as opposed to those above this range which indicated an above normal character maturity. Even if there was a distinct association between low character maturity (LCM) and number of reported ADHD and ASD symptoms, a number of individuals (about 8%) without any ADHD or ASD symptoms (i.e., no scores on a diagnostic tool) showed a LCM, while a substantial number of individuals with ADHD scores in the pathological range still showed an average character maturity (13).

Results such as these support the notion that character maturity is a dynamic concept varying in range from immature to at least normal maturity among both individuals with and without different forms of neurodevelopmental disorders. There is no reason to believe that the situation would be otherwise with regard to individuals with AAB, even if there is evidence for an association between TCI character dimensions and amount of AAB. A plausible supposition would be that subjects with low, medium, and high character maturity (HCM) would be found among these individuals, even if the majority would show a LCM.

### Current Study

In this study, we take special interest in character maturity as it is a strong candidate in the advancement of well-being and agency and a promising target for early intervention in order to prevent personality disorders and to reduce criminal propensity among high-risk children with early-onset conduct problems (19, 20). The development of character maturity appears to be related to a successful social adaptation to the progression over the lifespan, which is more marked among those low in character maturity (21, 22). The maturational processes among the latter would most probably benefit from treatment interventions as indicated by data from the Conduct Problems Prevention Research group (23). Particularly interventions directed against Self-Directedness would seem promising, since this factor has emerged as related to several aspects of well-being and a mature functioning regardless of how it interacts with other TCI dimensions (24).

The overall aim of this study is to investigate whether character maturity, as measured by the TCI dimensions Self-Directedness and Cooperativeness, is associated with AABs in young male offenders. More specifically, this study aims at exploring to what extent character maturity is related to (i) a diagnosis of ADHD, CD, and substance use disorder (SUD), (ii) type of criminal activity and overall level of persistence in criminality, (iii) expert assessed and self-rated measures of aggression, and (iv) psychopathic personality traits. Finally, it also aimed to investigate the role of character maturity on (v) overall occurrence of AABs/psychopathic traits when controlling for ADHD.

## Subjects and Methods

### Participants and Their Group Membership

Participants in this analysis (*N* = 148) were derived from a Swedish prison study of young male offenders convicted for hands-on violent and/or sexual criminality, *The Development of Aggressive Antisocial Behavior Study* (DAABS), investigating early-onset disruptive behavioral and mental disorders in a cohort of violent offenders. This study included young male offenders serving a prison sentence at a facility within the Western region of the Swedish Prison and Probation Services between March 2010 and July 2012. All male inmates aged 18–25 were asked to participate in the study, while inmates with insufficient language skills and those who were relocated before the data collection could be completed were excluded. Two hundred seventy male offenders gave informed consent among 380 possible participants, resulting in a 71% response rate. A more detailed presentation of this project is given by Wallinius and coauthors (25).

Among all participants in the DAABS, 148 male prisoners had given valid answers to Cloningers TCI (4, 26), consisting of 238 yes/no items measuring four temperament (i.e., Novelty Seeking, Harm Avoidance, Reward Dependence, and Persistence) and three character (i.e., Self-Directedness, Cooperativeness, and Self-Transcendence) dimensions. *T*-scores (with a mean of 50 and a SD of 10) were calculated for each dimension based on Swedish normative data (27), and the two character dimensions of Self-Directedness (SD) and Cooperativeness (Co) were used to identify character maturity. Those with a *T*-score more than 1 SD below the mean, that is a *T*-score below 40, on both character dimensions (SD and Co) were placed into the LCM group consisting of 43 male offenders with a mean age of 22.1 years (± 1.9), while those for which one of the two character dimensions were 1 SD below the mean and the other dimension within the normal range, that is a *T*-score between 40 and 60, were defined as the medium character maturity (MCM) group comprising 54 male offenders with a mean age of 21.7 years (± 1.9). Finally, all who at least reached a *T*-score within the normal range for both character dimensions (SD and Co), that is a *T*-score above 40 or more, constituted the HCM group encompassing 51 male offenders with a mean age of 21.6 years (± 1.9).

The group with valid TCI protocols (*n* = 148) were compared with those with invalid protocols (among which the number of available subjects varied between *n* = 122 and *n* = 60 due to missing values on single variables) for all studied variables (i.e., criminal history variables, DSM-IV diagnoses, and aggressive behavior and psychopathic traits) finding no significant differences between the two groups what so ever (data available upon request).

### Measures

#### Diagnostics of ADHD, CD, and SUD

DSM-IV (7) diagnoses of ADHD, CD, and SUD were established based on clinical interview data, according to the Structured Clinical Interview guide for axis I (28), and file information provided by the Swedish Prison and Probation Services. Number of fulfilled symptoms of ADHD was assessed for childhood as well as for adulthood and, in addition, categorical diagnoses were established, regardless of the presence of other diagnoses.

#### Criminal History

Data on criminal history covering previous and current criminality (index offense included) as well as age at onset of different forms of criminality was collected by means of a structured protocol that took both self-reported and file-based information into consideration. When self-reported and file-based information was contradictory, self-reported data were chosen as long as it appeared credible and reasonable. This made it possible to include data about criminal behavior and onset of criminality before the age of 15, since this is the age of criminal responsibility in Sweden, and thus the age from which files and sentence documents are available. Criminal behavior was divided into several different types, where any criminality (consisting of all kinds of criminal offenses), violent criminality (murder/manslaughter, assault, unlawful threat, robbery, and fire setting/arson), sexual criminality (all sexual acts according to the Swedish Penal Code), property criminality (stealing or destruction of property), and drug-related criminality are used in this study. Finally, persistence in criminality was based on total number of convictions, where all sentences for each participant were summed up and, thus, the higher the figure the more persistent in criminality.

#### Aggressive Antisocial Behavior and Psychopathic Traits

##### Aggression Questionnaire-Revised Swedish Version

Self-rated aggressive behaviors were captured by the Aggression Questionnaire-revised Swedish version (AQ-RSV) (29), which is a Swedish adaption of the Aggression Questionnaire (30). The Swedish version is a 29-item questionnaire where each item is answered on a 4-point Likert scale, ranging from “least characteristic” to “most characteristic.” AQ-RSV consists of the four subscales Physical aggression, Verbal aggression, Anger and Hostility, and one Total aggression score, where each scale score is the sum of its items and the total score is the total of all subscales. The AQ-RSV showed comparable psychometric properties to the original version, with alpha coefficients indicating considerable internal consistency for the AQ-RSV (30).

##### Life History of Aggression

Aggressive antisocial behaviors in a lifetime perspective were measured by the LHA (31), a questionnaire where 3 different types of aggressive and antisocial behaviors assessed by 11 items are rated on a 5-point scale based on the frequency of occurrences of the behavior in question from adolescence to the present (ranging from 0 = no events to 5 = so many events that they cannot be counted). The LHA consists of three subscales measuring aggression (including items covering temper tantrums, physical fights, verbal aggression, physical assaults on people or animals, and assaults on property), antisocial behavior (containing items about school disciplinary problems, problems with supervisors at work, and antisocial behavior with or without police involvement), and self-directed aggression (items concerning self-injurious behavior and suicide attempts). Finally, all items are summed up into a LHA total score. This instrument is characterized by excellent test–retest stability, interrater agreement, and internal consistency for both the LHA total score and the LHA aggression subscore, supporting the use of this instrument for especially the assessment of aggressive behaviors in a life-history perspective (32). Scale scores are computed by adding the items constituting each scale, rendering a total LHA scale score of 55. Total scale scores above 15 and/or an aggression subscale score above 12 are considered to indicate an abnormally high occurrence of lifetime aggressive behaviors. In this study, the LHA was administered as a clinician-rated instrument, where the assessor based the ratings on an evaluation of the interview as well as the file-based information.

##### Psychopathy Checklist-Revised

Psychopathic personality traits were assessed by the PCL-R (33). The PCL-R is developed to capture lifetime psychopathic personality traits, by 20 items that are rated on a 3-point scale (0 = does not apply, 1 = may apply or in some respects applies, 2 = does apply) and summed up to a total score ranging from 0 to 40. All available information, that is, from the interview, from observations during the assessment and from accessible files, was used for the assessment. Both the four-facet structure (interpersonal, affective, lifestyle, and antisocial) proposed by Hare and Neumann (34) and the original two factor model (Factor 1 encompassing the interpersonal and emotional features and Factor 2 the impulsive, irresponsible, and antisocial features) were used in the current analysis. Internationally, a score of 30 or above have been established as the cut-off for a highly elevated level of psychopathic traits. This has, however, been questioned with regard to European results were a total PCL-R score of 25 or above has been suggested as the cut-off signaling an elevated level of psychopathic traits (35).

### Statistics

Descriptive statistics in the form of mean values, SDs, and frequency or percent are used for presenting the studied variables. Group differences between the three groups with LCM, MCM, and HCM were analyzed by one-way between groups analysis of variance (ANOVA) for continuous variables, and by Chi-square tests and Fisher’s exact test (when any cell count were less than five) for dichotomous variables. Levenes’s test for equality of variance were used to check for violations of the assumption of homogeneity, that is, that the variance in the used variables is the same for each of the three groups, and thus used as a guide to choose the proper *post hoc* test (Tukey versus Tamhane’s T2) to identify significant group differences when the ANOVA showed a between groups effect. Similarly, a standardized residual equal to or above ±1.96 were used as a measure to decide which cell contributed to the effect when the Chi-square or Fisher’s exact test showed a significant association. In addition, measures of association have been used as expressions of effect sizes, where η^2^ was used for the analyses carried out by an ANOVA, and Ф coefficient for the cross tabulations analyzed by Chi-square tests or Fisher’s exact test. Effect sizes were interpreted according to common recommendations (36) stating that for Ф a value of 0.10 is equivalent to a small effect, 0.30 to a medium, and 0.50 to a large effect, while for η^2^ a value of 0.01 corresponds to a small effect, 0.06 to a medium, and 0.14 to a large effect.

Finally, a one-way between groups multivariate analysis of covariance (MANCOVA) were performed to investigate whether character maturity, over and above the effect of ADHD, were related to aggressive behaviors and psychopathic personality traits. Total scale scores of AQ-RSV, LHA, and PCL-R were used as dependent variables, while character maturity as measured by TCI were used as the independent variable, whereas childhood ADHD in the form of number of fulfilled DSM-IV criteria for an ADHD diagnosis was controlled for. Preliminary assumption testing was conducted, checking for reliability of covariate measure, normality, linearity, univariate and multivariate outliers, homogeneity of variance–covariance matrices, homogeneity of regression, and multicollinearity, with no serious violations except for equality of variance for the variable LHA total scores. Due to this violation, as for the repeated comparisons, a more conservative alpha level of 0.001 for determining significance in the univariate *F*-test was chosen. For the multivariate test of significance, Pillais’ Trace was chosen in favor for the more common Wilk’s Lambda, due to the fact that the former is a more robust test and thus more appropriate when some of the assumptions are not quite satisfied.

### Ethics

This study was approved by the Research Ethics committee at the Lund University (Dnr: 2009/405). All participants consented to participation after having received both written and oral information about the study. Participants were rewarded with 200 SEK (equal to about 25$), substituting for the eventually loss of payment due to missed participation in the prison activity during the day of assessment. The amount of the monetary reward, small enough to not create an incentive that would compromise the free ground for participating in the study, was approved by the Ethics committee. Finally, participants who showed signs of severe psychopathology were given the opportunity to be referred to the prisoner psychiatrist for further assessment and treatment whenever there was such an option.

## Results

### Character Maturity, ADHD, CD, and SUD

The results of the statistical analyses of ADHD, CD, and SUD variables are presented in Table 1. An association with a medium effect size was found for character maturity and ADHD, with most individuals fulfilling criteria for a diagnosis both during childhood as well as in adulthood in the group with the lowest character maturity, and correspondingly the fewest among the group highest in character maturity. A similar pattern with medium effect sizes were found for occurrence of a CD and a SUD diagnosis, where the LCM group had the largest percentage of those diagnosed and the HCM group had the smallest, although the overall percentage of those diagnosed were high in all three character groups.

**Table 1:** **Prevalence of ADHD, CD, and SUD (any substance) diagnoses in the three groups of low, medium, and high character maturity**.

A DSM-IV diagnosis of ADHD, CD, or SUD (any substance)	LCM (%)	MCM (%)	HCM (%)	χ^2^ or Fisher’s exact test	*p*-Value	Φ
ADHD during childhood, *n* (%)				15.76	≤0.001	0.33
Yes	36 (83.7)	37 (68.5)	23 (45.1)
No	7 (16.3)^a^	17 (31.5)	28 (54.9)^a^
ADHD in adulthood, *n* (%)				20.25	≤0.001	0.37
Yes	28 (65.1)^a^	25 (46.3)	10 (19.6)^a^
No	15 (34.9)^a^	29 (53.7)	41 (80.4)^a^
CD, *n* (%)				16.29	≤0.001	0.34
Yes	40 (95.2)^a^	46 (85.2)	32 (62.7)^a^
No	2 (4.8)	8 (14.8)	19 (37.3)
SUD (any substance), *n* (%)				23.93	≤0.001	0.39
Yes	43 (100)	48 (88.9)	33 (64.7)
No	0 (0)^a^	6 (11.1)	18 (35.3)^a^

*LCM, low character maturity; MCM, medium character maturity; HCM, high character maturity; ADHD, attention-deficit hyperactivity disorder; CD, conduct disorder; SUD, substance use disorder*.

*^a^Standardized residual ≥±1.96*.

### Character Maturity and Criminal History

In Tables 2 and 3, the results of the statistical analyses of criminal history are presented. A significant negative association of medium strength was overall found between character maturity and criminal history variables that indicate severity of criminality, such as age at onset of both any and violent criminality, persistence in criminality, and previous drug-related criminality. A somewhat weaker association was found for the remaining variables covering both previous violent, sexual, and property criminality as well as violent and sexual index criminality. For all variables except sexual criminality the associations went in the direction of less criminality or later age at onset the higher the character maturity. For sexual criminality, the association went in the opposite direction, with the highest amount of individuals with both previous sexual criminality and a sexual index crime in the HCM group.

**Table 2:** **Age at onset of any criminality and of violent criminality, as well as number of prior violent criminal acts in the three groups of low, medium, and high character maturity**.

	LCM	MCM	HCM	*F*	*p*-Value	η^2^	Post hoc test
**Age at onset of criminality**							
Any criminality, mean (SD)	12.47 (3.47)	13.11 (3.62)	15.14 (4.84)	5.723	≤0.01	0.07	LCM < HCM**
	*n* = 43	*n* = 53	*n* = 50				
Violent criminality, mean (SD)	16.21 (3.43)	15.83 (3.34)	18.04 (3.65)	5.74	≤0.01	0.08	LCM < HCM*
	*n* = 42	*n* = 53	*n* = 50				MCM < HCM**
**Persistence in criminality**							
Number of convictions, mean (SD)	5.93 (5.28)	4.87 (3.65)	2.55 (2.99)	8.87	≤0.001	0.11	LCM > HCM**
	*n* = 42	*n* = 52	*n* = 51				MCM > HCM*

*LCM, low character maturity; MCM, medium character maturity; HCM, high character maturity*.

*p ≤ 0.05.

**p ≤ 0.001.

**Table 3:** **Previous criminality and index criminality in the three groups of Low, Medium and High character maturity**.

	LCM (%)	MCM (%)	HCM (%)	χ^2^ or Fisher’s exact test	*p*-Value	Φ
**Previous criminality**						
Violent criminality, *n* (%)				1.78	n.s.	0.11
Yes	43 (100)	54 (100)	50 (98.0)			
No	0 (0)	0 (0)	1 (2.0)			
Sexual criminality, *n* (%)				7.44	≤0.05	0.24
Yes	3 (7.0)	5 (9.3)	13 (25.5)^a^			
No	40 (93.0)	49 (90.7)	38 (74.5)			
Drug-related criminality, *n* (%)				29.04	≤0.001	0.42
Yes	40 (93.0)	42 (77.8)	24 (47.1)^a^			
No	3 (7.0)^a^	12 (22.2)	27 (52.9)^a^			
Property criminality, *n* (%)				5.44	n.s.	0.20
Yes	40 (93.0)	48 (88.9)	39 (76.5)			
No	3 (7.0)	6 (11.1)	12 (23.5)			
**Index criminality**						
Violent criminality, *n* (%)				4.41	n.s.	0.18
Yes	41 (95.3)	49 (92.5)	42 (82.4)			
No	2 (4.7)	4 (7.5)	9 (17.6)			
Sexual criminality, *n* (%)				6.01	≤0.05	0.21
Yes	3 (7.0)	5 (9.4)	12 (23.5)^a^			
No	40 (93.0)	48 (90.6)	39 (76.5)			

*LCM, low character maturity; MCM, medium character maturity; HCM, high character maturity*.

*^a^Standardized residual ≥ ± 1.96*.

### Character Maturity and Aggressive Antisocial Behaviors

The results for the AABs, both with regard to self-rated AQ-RSV scale scores and to expert-rated LHA scale scores, are presented in Table 4. A rather consistent pattern emerged irrespective of whether AABs were self-rated or assessed by a clinical expert, with the highest scale scores in the LCM group, and decreased scores following higher character maturity. For almost all scales (the only exception was found for the LHA Self-directed aggression where the only significant difference was found between the LCM group that showed more self-harm than the MCM group), significant differences were found between the HCM group and the two groups with LCM and MCM. Total scale scores for both the AQ-RSV and the LHA showed a pattern with a stepwise decrease in scores from highest in the LCM group to lowest in the HCM group, with each group significantly differing from all the other groups. The association between character maturity and the different measures of AABs were, except for LHA Self-directed aggression, within the range of a medium to a large effect size.

**Table 4:** **Aggression Questionnaire-Revised Swedish Version scale scores (mean, SD), Life History of Aggression scale scores (mean, SD), and psychopathy checklist-revised scale scores (mean, SD) for the three groups of low, medium and high character maturity**.

	LCM	MCM	HCM				
					
AQ-RSV	(*n* = 42)	(*n* = 53)	(*n* = 51)	*F*	*p*-Value	η^2^	Post hoc test
Total Aggression score, mean (SD)	108.79 (16.96)	98.53 (18.47)	83.96 (20.07)	20.98	≤0.001	0.23	LCM > MCM*
							LCM > HCM***
							MCM > HCM***
Physical aggression, mean (SD)	36.62 (6.82)	35.21 (6.81)	30.22 (8.87)	9.47	≤0.001	0.12	LCM > HCM***
							MCM > HCM**
Verbal aggression, mean (SD)	17.83 (3.63)	17.07 (3.50)	14.75 (3.52)	9.94	≤0.001	0.12	LCM > HCM***
							MCM > HCM**
Anger, mean (SD)	26.07 (5.41)	22.79 (6.18)	19.55 (6.79)	12.97	≤0.001	0.15	LCM > MCM*
							LCM > HCM***
							MCM > HCM*
Hostility, mean (SD)	28.38 (5.92)	23.30 (7.21)	19.45 (6.31)	21.40	≤0.001	0.23	LCM > MCM**
							LCM > HCM***
							MCM > HCM**

**LHA**	**(*n* = 43)**	**(*n* = 54)**	**(*n* = 51)**				

Total score, mean (SD)	36.67 (6.99)	31.24 (8.91)	24.10 (10.83)	22.46	≤0.001	0.24	LCM > MCM*
							LCM > HCM***
							MCM > HCM***
Aggression, mean (SD)	20.65 (4.24)	17.22 (5.61)	14.22 (6.30)	15.91	≤0.001	0.18	LCM > MCM**
							LCM > HCM***
							MCM > HCM*
Antisocial behavior, mean (SD)	14.63 (2.98)	13.56 (3.89)	9.31 (4.97)	23.20	≤0.001	0.24	LCM > HCM***
							MCM > HCM***
Self-directed aggression mean (SD)	1.40 (2.34)	0.46 (1.49)	0.57 (1.30)	4.02	≤0.05	0.05	LCM > MCM*

**PCL-R**	**(*n* = 43)**	**(*n* = 52)**	**(*n* = 51)**				

Total score, mean (SD)	19.15 (5.42)	20.63 (6.83)	13.96 (8.22)	12.75	≤0.001	0.15	LCM > HCM**
							MCM > HCM***
Factor 1 (interpersonal affective), mean (SD)	3.67 (2.85)	5.21 (3.29)	3.63 (3.29)	4.08	≤0.05	0.05	MCM > HCM*
Factor 2 (antisocial lifestyle), mean (SD)	14.79 (3.83)	14.52 (4.38)	9.63 (5.76)	18.38	≤0.001	0.20	LCM > HCM***
							MCM > HCM***
Facet 1 (interpersonal), mean (SD)	0.74 (0.98)	1.06 (1.35)	0.96 (1.50)	0.69	n.s.	0.01	
Facet 2 (affective), mean (SD)	2.93 (2.25)	4.15 (2.39)	2.67 (2.29)	5.98	≤0.01	0.08	MCM > LCM*
							MCM > HCM**
Facet 3 (lifestyle), mean (SD)	7.42 (2.07)	7.29 (2.15)	4.94 (2.82)	16.92	≤0.001	0.19	LCM > HCM***
							MCM > HCM***
Facet 4 (antisocial), mean (SD)	7.37 (2.28)	7.23 (2.68)	4.68 (3.25)	14.62	≤0.001	0.17	LCM > HCM***
							MCM > HCM***

*LCM, low character maturity; MCM, medium character maturity; HCM, high character maturity; AQ-RSV, Aggression Questionnaire-Revised Swedish Version; LHA, life history of aggression; PCL-R, psychopathy check list-revised*.

*p < 0.05.

**p < 0.01.

***p < 0.001.

### Character Maturity and Psychopathic Personality Traits (PCL-R)

Data concerning psychopathic personality traits according to the PCL-R are presented in Table 4. For PCL-R total score both the LCM and the MCM groups showed significantly higher scores than the HCM group. When comparing PCL-R factors, the MCM group showed a significantly higher score than the HCM group for PCL-R Factor 1, while both the LCM and the MCM groups displayed significantly higher scores than the HCM group for Factor 2. A more detailed pattern of differences and similarities emerged when comparing the four facets of PCL-R. It was only Facet 2 of the two facets belonging to Factor 1 that emerged as significant with the MCM group higher than the two other groups (LCM and HCM). For the more behaviorally oriented Facet 3 and 4 a similar pattern emerged, since the two groups of LCM and MCM showed significantly higher scores in both cases than what was found for the HCM group. Effect sizes for all scales where a significant association were found indicated most often a strong relationship (i.e., a large effect size) between character maturity and psychopathic personality traits. Exceptions from this pattern were found for PCL-R Facet 2 and PCL-R Factor 1 for which the effect sizes lied in the medium range.

### The Overall Effect of Character Maturity on Aggressive Antisocial Behaviors and Psychopathic Personality Traits When Controlling for ADHD

**Figure 1:**
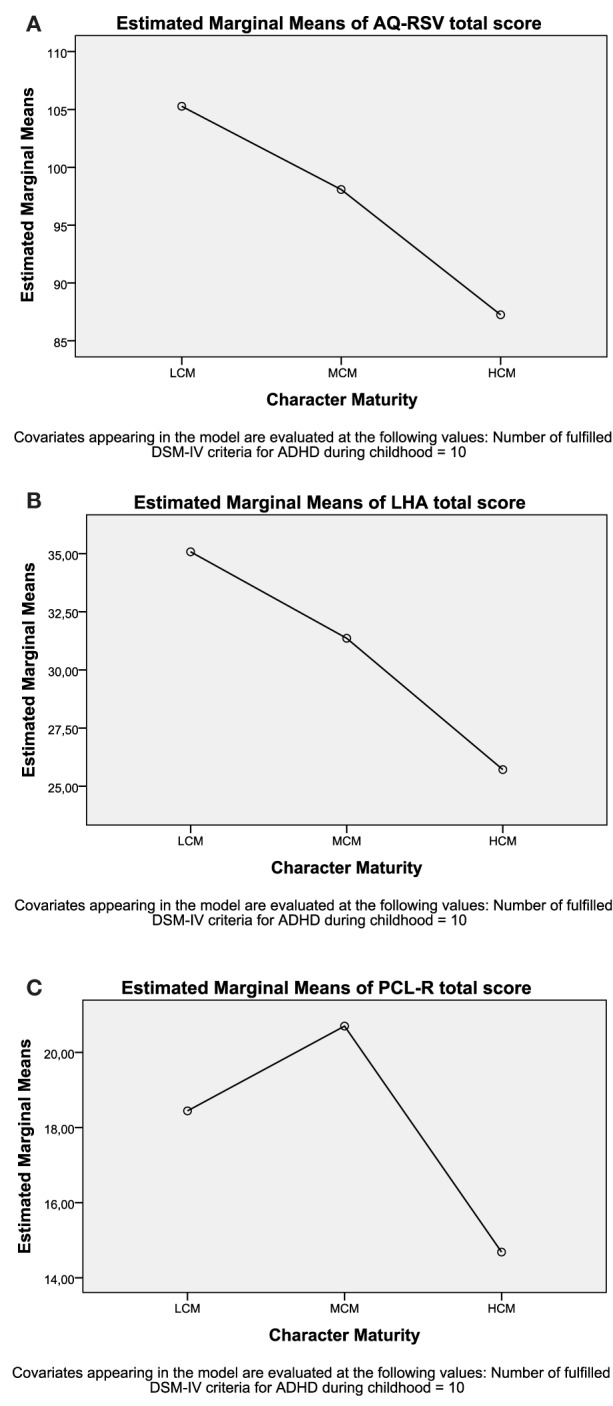
**(A–C)** Illustration of estimated marginal means for the variables of AQ-RSV total score, LHA total score, and PCL-R total score for the three groups of different character maturity with covariate (number of fulfilled DSM-IV criteria for ADHD = 10) appearing in the model.

There was, according to the MANCOVA, a statistically significant difference with regard to both the covariate number of fulfilled DSM-IV ADHD diagnostic criteria and the three character maturity groups (LCM, MCM, and HCM) on the combined dependent variables (i.e., aggressive behaviors and psychopathic traits). For the covariate, the MANCOVA showed the following values; *F* (3, 137) = 9.02, *p* < 0.001; Pillai’s Trace = 0.17; partial η^2^ = 0.17 and, for character maturity, the corresponding values were; *F* (6, 276) = 6.83, *p* < 0.001; Pillai’s Trace = 0.26; partial η^2^ = 0.13. When results for the dependent variables were considered separately, significant differences for number of fulfilled DSM-IV diagnostic criteria for ADHD, using an adjusted alpha level of *p* ≤ 0.001, were found for AQ-RSV total score, *F* (1, 139) = 20.80, *p* < 0.001; partial η^2^ = 0.13 and for LHA total score, *F* (1, 139) = 20.90, *p* < 0.001; partial η^2^ = 0.13. For character maturity, significant differences were found for all three variables; AQ-RSV total score, *F* (2, 139) = 10.75, *p* < 0.001; partial η^2^ = 0.13, LHA total score, *F* (2, 139) = 12.09, *p* = < 0.001; partial η^2^ = 0.15, and PCL-R total score, *F* (2, 139) = 9.16, *p* < 0.001; partial η^2^ = 0.12. An inspection of the mean scores with the covariate number of fulfilled DSM-IV diagnostic criteria appearing in the model, evaluated at the value of 10 fulfilled criteria, indicated that there was a linear relationship between measures of aggression (i.e., AQ-SRV and LHA) and character maturity (see Figures 1A–C), with the highest scores for the LCM group (105.3 and 35.1, respectively) and then descending showing the lowest scores for the HCM group (87.3 and 25.7, respectively). However, psychopathic personality traits (i.e., PCL-R total scores) deviated from this trend (see Figures 1A–C) showing the highest scores (20.7) for the MCM group and lower scores for both the LCM group and the HCM group (18.4 and 14.7, respectively).

## Discussion

This study investigated the relation between character maturity assessed by Cloninger’s TCI and AAB, with special emphasis on measures of aggression and psychopathic personality traits, in a nationally representative cohort of young Swedish violent offenders. In a first step, we divided these offenders into three groups based on level of character maturity (i.e., low, medium, and high in maturity) and found a rather consistent pattern where level of character maturity was related to outcome such as that the lower the maturity level the more of externalizing disorders (i.e., ADHD, CD, and SUD) and AABs. The only measure that clearly diverged from this pattern was found for sex crimes, where the highest level of character maturity was related to the most frequent occurrence of this type of criminality. In a second step, we analyzed whether character maturity was related to AABs and psychopathic traits when controlled for ADHD, and found that the same pattern as described above emerged even though we controlled for number of fulfilled DSM-IV criteria of ADHD. Thus, character maturity emerged as a factor that seemed to be strongly related to varied measures of AAB, in the, to our knowledge, first study to investigate these aspects in violent offenders.

One of the strongest and most consistent associations between character maturity and the studied outcome variables was found for the three disorders of ADHD (both during childhood, and in adulthood), CD, and SUD, with a stepwise reduction of number of individuals for each of these diagnoses the higher the level of character maturity. The effect sizes of these associations were all in the upper part of the medium range, supporting a rather distinct pattern where LCM frequently is associated with externalizing disorders (i.e., ADHD, CD, and SUD). This resembles what other researchers have found [e.g., Ref. (13, 18, 37)], and thus confirms the picture of an relationship between low Self-Directedness and Cooperativeness, that is, LCM, in individuals with neurodevelopmental disorders, and also that when these disorders are seen in combination with AABs such as seen in ODD and CD, even higher levels of behavioral and emotional difficulties are found. Though a cross-sectional design is not ideal to draw conclusions on developmental pathways, a tentative suggestion would be that the neurodevelopmental aberrations underlying ADHD, in combination with an early onset of aggression, “normlessness” and subsequently substance abuse, that is, early-onset externalizing disorders, hampers the maturational processes and makes it difficult, but not impossible, to develop a functional character. Hence, many but not all offenders with these problems present with LCM, and there is independent of occurrence of disorder a number of individuals with normal to high character maturity.

There was also an association between level of character maturity and criminal behaviors, but this relation was not as clear as was the one found for ADHD, CD, and SUD. For variables such as onset of any criminality or violent criminality, as well as persistence in criminality, it was the HCM group that significantly separated itself from the other two groups. Thus, the HCM group was distinguished by a later onset of both any and violent criminality, and likewise with the lowest mean number of sentences suggesting a less persistent tendency to develop AABs. This pattern resembles Moffitt’s taxonomy (1) of adolescence-limited versus life-course persistent AAB, since it more or less could be described as a demarcation line separating the HCM group from the other two groups who are characterized by an early onset and frequent acts of violence. Instead of a stepwise association between character maturity and onset of criminality or frequency of violent acts, it appears to be related in a more absolute way indicating that character maturity must have reached a certain level of maturity before it makes any difference with regard to criminality. This is most probably especially relevant with regard to Self-Directedness, which, according to Cloninger (5), corresponds to the ability to supervise or execute behavior, or in other words, to initiate, regulate, and organize behavior toward voluntary goals in accordance with the surrounding social context.

One type of criminality that significantly deviated from the otherwise dominant pattern of violent criminality was sex crimes, which somewhat surprisingly were most frequent in the HCM group. Overall, sex crimes were quite unusual in the LCM and MCM, occurring only in about 7–9% percent of these individuals, while it was found for almost 24% of those in the HCM group. A tentative interpretation of this pattern would be that sexual criminality is more frequent in mature individuals and that it might follow a separate trajectory distinct from common forms of antisocial behaviors. Almost all of the sex offenders were acquainted to or in a relation with their victims (half committed rape against adults and a third against children). The fact that almost every sex offender knew their victims in one way or another would indicate an ability to interact and create a positive mutual relationship for at least some period of time, and relations need some measure of maturity or at least an understanding of how a social bond is established and maintained (i.e., cooperativeness), why a premise of this kind of crime most probably is a certain level of character maturity. In the ongoing debate about whether sex criminals specialize in sexual offending or if their sex crimes are an expression of their general antisocial behavior leading to a broad array of offenses, our findings also corresponds to some extent with a recent study stating that the rather limited group of individuals specialized in sexual criminality are characterized by a better mental health (i.e., less likely to have received psychiatric or mental health counseling) than what is seen in versatile sex offenders (38).

A consistent, significant pattern was also seen for the relation between aggression and character maturity. Both self-rated and expert-rated measures for aggression were for a majority of the used scales inversely associated with character maturity; the higher the level of maturity the lower the value for the different measures of aggression. The effect size was large for a majority of these associations regardless of if they were self- or expert-rated, indicating that there is a rather strong relation between level of aggression and character maturity. While the self-rated aggression measures could be expected to reflect more of a state position, the contrary could be said about the expert-ratings since they assessed level of aggression in a life perspective. It is interesting to see that these two sets of assessments corresponds so well to each other, giving rise to a unified picture suggesting that the impact of character maturity on the self-perception of aggression and anger as well as the occurrence of aggressive acts over the life-course is of significant importance. Since this is the first attempt to unveil the relation between aggression and character maturity in a male violent offender sample the need for replication in other offender samples is pressing. There is, however, support for a relation between the character dimension of low Self-Directedness and anger from studies on subjects with an anger syndrome (39), and with attempted suicide (40), while aggressive behavior have been related to low Self-Directedness in children with Child Behavior Checklist-Dysregulation (41). These results, taken together with ours, add to the picture of character maturity as a vital psychological construct of importance for the experience of anger and aggression and the ability to control and regulate aggressive reactions.

To judge from the results in this group of young male violent offenders the relation between psychopathy as measured by the PCL-R and character maturity as composed of Self-Directedness and Cooperativeness appeared as somewhat complex, and to some extent independent of level of character maturity. There were no differences in Facet 1 (interpersonal) between the three character maturity groups, while the highest scores for Facet 2 (affective) were found in the MCM group. For the more behaviorally oriented Facets 3 and 4 (lifestyle, and antisocial), the HCM group significantly differed from the other two groups showing the lowest scores. Changing perspective from Facets to PCL-R Factors 1 and 2 did for obvious reasons not change the pattern, why in Factor 1 the MCM group significantly differed from the HCM group. In Factor 2 as well as for PCL-R total score, the HCM group showed the significantly lowest scores compared to the other two groups. The latter resembles the pattern we saw in the relation between criminality and character maturity, which is hardly surprising since Factor 2 largely is composed of items related to criminality and criminal behaviors. Our results concerning psychopathy and its relation to character maturity, that is Self-Directedness and Cooperativeness, did not resolve the to some degree contradictory results that was seen between other studies on this topic (8–10). One plausible interpretation of this situation is that character maturity is associated with the behavior components of psychopathy, that is, impulsive, irresponsible, and criminally colored behaviors since they are dependent on the individuals ability to regulate and execute volitional behavior, but not with the personality components of this construct, that is, the narcissistic and affective traits. Our findings would then support the notion that psychopathy is a separate aspect of personality that is in part unrelated to what is considered normal character formation.

To further investigate the association between character maturity on the one hand and aggression and psychopathy on the other, a MANCOVA with total scale scores of AQ-RSV, LHA, and PCL-R were used as dependent variables while at the same time the effect of ADHD were controlled for. Low Self-Directedness and low Cooperativeness have been a nearly constant finding in research with TCI on individuals with ADHD [e.g., Ref. (13, 15, 18)], why there is reason to ensure that the association between character maturity and measures of criminality and aggression seen in our group of male violent offenders not merely was an artifact of the high occurrence of individuals with ADHD in this group. The results of this analysis strengthened the picture of character maturity as associated with aggressive behaviors since it clearly showed a stepwise association over and above what could be attributed to an effect of ADHD. Thus, character maturity appeared as an independent factor that in itself emerged as strongly related to both self- and expert-rated aggressive behaviors, where a HCM acted as a protective quality leading to a reduction in total scale scores on the AQ-SVR and LHA instruments. Psychopathy, on the other hand, did not emerge as associated with character maturity in a stepwise or linear way when ADHD was controlled for. Instead this analysis confirmed the pattern found in the univariate analyses, indicating that character maturity must reach a certain level of development for to have any counteracting or decreasing effects on PCL-R total scores as a measure for psychopathic personality traits.

### Limitations

Studies on humans are usually affected by limitations and this study is no exception. First of all, our study group consists only of male offenders due to the fact that inclusion of study subjects occurred on a regional basis, from which it was not possible to recruit a scientifically motivated large enough number of female offenders. Second, this study group is a subsample derived from a larger cohort of young male violent offenders, based on those who had valid TCI protocols thus possibly indicating some bias, even if there were no differences between those with valid protocols compared to those without in the studied variables. Furthermore, this study group is also a fairly homogenous group of violent offenders (i.e., they were included due to violent criminality), which limited the variance with regard to violent criminality and hampered the possibility to study the effects of character maturity on the propensity for violent criminality. However, our findings, even if the number of studied subjects was somewhat restricted and of a homogeneous art, were rather consistent and in many cases associated with a large effect. Another aspect concerns the fact that several variables regarding previous criminality are based on self-reports not possible to compare to official records or sentences. However, research regarding accuracy in self-reports has found that it in many aspects are representative of the actual crime history only marginally giving rise to underreporting, and that self-reported age at onset are a few years earlier compared to official records, and thus self-reported data could be viewed as quite reliable (42). On the other hand, since any limitation due to self-reported data applies with equal strength to all three groups, would its impact on comparisons between these groups would be negligible. All in all, generalizations from these results should be made with some caution and restricted to young male violent offenders from countries with similar rates of criminality as in Sweden.

### Conclusion

Based on our data, character maturity, as measured by the TCI dimensions Self-Directedness and Cooperativeness, emerged as a probable protective factor since almost all measures of AABs decreased with higher levels of character maturity. This was especially true for SUD where all offenders with the lowest character maturity fulfilled criteria for a DSM-IV SUD diagnosis, while offenders with SUD decreased, in a stepwise mode, with growing character maturity. Substance use is a major factor behind crime and violence (43, 44) and as such one of the most important factors for risk management to get hold of. Our results concerning the relation between character maturity and substance abuse as well as its relation to AABs, identify character maturity as one of the more potential psychological aspects that should be targeted for treatment interventions, with a possibility to decrease the intensity and incidence of both substance use and AABs.

Our results are not the only to focus on character maturity (i.e., Self-Directedness and Cooperativeness) as a psychological aspect related to a positive development in humans. High Self-Directedness and Cooperativeness has also emerged as strongly correlated with resilience and capacity to handle stress in both men and women (45), and furthermore as important determinants of well-being (especially with regard to Self-Directedness) (46). Cloninger concludes in a recent research overview that the most happy, healthy, and fulfilled individuals are those who are high in the TCI character dimensions (47). Character maturity as described by the TCI dimensions of Self-Directedness and Cooperativeness thus holds great promise as targets for therapeutic interventions, especially since they are constructs covering executive and regulating functions of personality that seems to have the potential to develop as a response to empathic supervision, training, and supportive counseling. Therapeutic interventions of this kind should particularly be directed against children with early-onset externalizing disorders in combination with AABs aiming at reversing the development in a positive direction with the goal to prevent the emergence of substance abuse and criminal activity. To conclude, character maturity emerged as strongly related to AABs, and it is a prominent candidate for treatment interventions that calls for the development of targeted programs that are subjected to systematic evaluations.

## Author Contributions

TN conceived the study and drafted the manuscript together with ÖF. TN, ÖF, and BH designed the study, while EB, MW, and BH obtained the data. TN and ÖF did all the analyses. All the authors critically revised the manuscript and approved the final version.

## Conflict of Interest Statement

The authors declare that the research was conducted in the absence of any commercial or financial relationships that could be construed as a potential conflict of interest.
